# A Biomimetic Lignocellulose Aerogel-Based Membrane for Efficient Phenol Extraction from Water

**DOI:** 10.3390/gels10010059

**Published:** 2024-01-12

**Authors:** Peipei Liu, Chunling Zheng, Zhong Yao, Fang Zhang

**Affiliations:** College of Food Science and Light Industry, Nanjing Tech University, Nanjing 211800, China; lppliupp@163.com (P.L.); zhengchunling@njtech.edu.cn (C.Z.)

**Keywords:** biomimetic membrane, cellulose–lignin aerogel, phenol, extraction and enrichment

## Abstract

Rapid extraction and concentration systems based on green materials such as cellulose or lignin are promising. However, there is still a need to optimize the material properties and production processes. Unlike conventional cellulose or lignin sorbent materials, aquatic reed root cells can concentrate external organic pollutants in the water and accumulate them in the plant. Inspired by this, a new nanocellulose–lignin aerogel (NLAG) was designed, in which nanocellulose was used as a substrate and lignin and polyamide epoxy chloropropane were used to crosslink cellulose in order to enhance the strength of the NLGA, resulting in good mechanical stability and water–oil amphiphilic properties. In practical applications, the organic membrane on the NLAG can transport organic pollutants from water to the NLAG, where they are immobilized. This is evidenced by the fact that the aerogel can remove more than 93% of exogenous phenol within a few minutes, highly enriching it inside. In addition, the aerogel facilitates filtration and shape recovery for reuse. This work establishes a novel biopolymer–aerogel-based extraction system with the advantages of sustainability, high efficiency, stability, and easy detachability, which are hard for the traditional adsorbent materials to attain.

## 1. Introduction

As green materials, cellulose and lignin of aquatic plant origin have the advantages of being environmentally friendly and sustainable, and they are often used to make separators or adsorbents [1]. However, the sorption performance and extraction concentration efficiency of cellulose and lignin materials are low, and there is an upper limit to the improvements in performance that can be achieved by simply relying on derivatives or changing their presentation [2,3]. In contrast, we found that aquatic plants have a complete bioextraction system for the purification of organic pollutants in water, which is much more efficient than using materials from the plants themselves or their derivatives [4,5]. The reed wetland is known as nature’s “second forest” due to its ecological value. The reeds’ root cells absorb and enrich organic matter from the water to purify it. This system of transporting organic pollutants from the “cell wall” to the “inner cell” of the reed cell has inspired us to purify water in a similar manner. While it is ideal to mimic a cellular structure based on green materials to extract organic pollutants efficiently, mimicking the cellular microstructure artificially is challenging. First, cellular nanostructures operate at the micro scale, posing a challenge in experimental manipulation. Second, to mimic the “cell wall” structure outside the plant cell aerogel, this structure must be of the right size and porosity to absorb and contain the organic pollutants.

On top of the above, plant cell biomimetic aerogels should be surrounded by a membrane to transport organic pollutants, and because molecules transfer faster in liquids than in solid membranes, oil–water liquid membranes can be considered to be the most effective separation technique. In this case, compatibility between the aerogel and the oil membrane is a prerequisite. In addition, the harsh environment that accompanies the preparation, extraction, and regeneration process requires that the device be sufficiently robust. However, this is extremely demanding in terms of the mechanical strength, shape, microstructure, and other properties of the device, which are difficult to achieve by simply extracting the material from the plant. For spherical materials, a variety of advanced properties and functions (such as water–oil (W/O) interfacial affinity, homogeneous surface properties, and reactivity) are critical, which are required for aerogels in energy, environmental, and biomedical applications [6]. The preparation of many porous structures often requires freeze-drying methods for structural modulation [7,8], and the highly porous structure of nano-phytobiont aerogels produced by such methods can accommodate large amounts of organic contaminants. However, in water, the porous structure tends to cause the aerogel to lose its mechanical strength, making it struggle to withstand experimental-treatment-induced stresses such as those arising during high-speed agitation, extrusion, and centrifugation. This hinders the practical application of porous structures in water treatment. In the natural environment, trees have a pristine form of lignin that acts as a cellulose binder—a substance that enhances the ability of trees to withstand harsh environments. Previously, we reported a simple lignin–polyamide epoxy resin nanopolymer for reinforcing cellulose paper and porous foam, with which the cellulose paper demonstrated ultrahigh wet strength [9,10]. Moreover, the aromatic polymer lignin is rich in hydroxyl groups, which have good hydrophilicity, and this substance is essential in the hydrophobic modification of cellulose and has been shown to favor the hydrophilic and hydrophobic interfacial compatibility [11] and to increase the stability of the nanocellulose composites [12,13]. The microscale structure and mechanical stability properties of the hypothetical plant cell biomimetic membrane system can be achieved with nanocellulose and lignin as the base materials of the aerogel, with the factors considered above.

In the present work, a new extraction system consisting of a nanocellulose polymerized lignin microdevice and an oil membrane layer is proposed, inspired by reeds in nature. In this system, the aerogels were prepared by means of ultrasonic atomization and the thermally driven crosslinking of lignin–polyamide epichlorohydrin (PAE) resin nanoaggregates with nanocellulose. After crosslinking, the aerogel can have a porous structure and, at the same time, ensure mechanical stability, because the functional groups of the material itself also have good affinity for the oil–water interface. Because of this feature, nanocellulose aerogels loaded with NaOH solution can be easily and uniformly dispersed in the oil phase to form biomimetic extracted cellulose aerogels, where organic contaminants in water are quickly absorbed by the aerogels through the oil membrane. The biomimetic extracted cellulose aerogels demonstrate super-efficient organic pollutant extraction and enrichment performance compared to the original cellulose or lignin sorbents, and the aerogels are easily filtered from the system, showing significant advantages in disassembly. In the present work, we attempted, for the first time, to establish a high-performance extraction and enrichment aerogel system to mimic the extraction mechanism of reed cells, which is impossible when using conventional cellulose or lignin sorbent materials.

## 2. Results and Discussion

**Figure 1:**
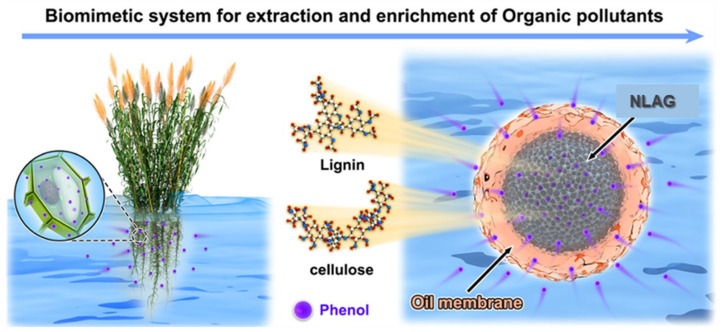
Schematic diagram of the biomimetic system for extracting and enriching organic pollutants from water: (**left**) reed root cell wall as a semi-permeable membrane for absorbing organic pollutants from water into its body; (**right**) the receiving core is NLAG, and the shell membrane is an oil phase for extracting organic pollutants from external water into the biomimetic aerogel.

In nature, most plant cells can remove and concentrate organic contaminants in water. As “nature’s kidney”, reeds protect the Earth’s precious water resources, and their root systems absorb organic, toxic pollutant compounds from wastewater [14]. Within the reed root system, the process of organic pollutant accumulation includes the steps of organic pollutant uptake by the root cell wall, transport of organic pollutants from the cell wall into the inner cell, cellular compartmentalization, and organic pollutant uptake and sequestration (Figure 1). Reeds have a high reproductive capacity and are found along rivers, lakes, ponds, ditches, and low wetlands. Apart from forest habitats, where they do not grow, various open areas with water sources often form continuous reed communities with their rapidly expanding reproductive capacity. This has led to attempts to explore the use of green materials to mimic the cell structure separation systems of replacement plants, of which nanocellulose and lignin have the most potential for establishing cell structure separation systems. Figure 1 shows that the nanocellulose–lignin aerogel (NLAG) and the encapsulated oil membrane layer form a cell-like separation system. The NLAG has a porous structure that can accommodate organic contaminants, while the oil membrane layer is like the cell wall membrane of reed roots. In addition to the structure, the mechanism of organic matter transport was also inspired by reed root cells. In plant cells, organic matter uptake proteins remove organic contaminants through ion exchange. The stripping agent (NaOH) in the biomimetic membrane system corresponds to the organic matter uptake protein and the organic matter efflux protein in the plant cell. The transfer of material between these two proteins is based on ion exchange. This intelligent extraction system allows organic contaminants to be selectively extracted by the oil membrane and trapped in the biomimetic aerogel.

**Figure 2:**
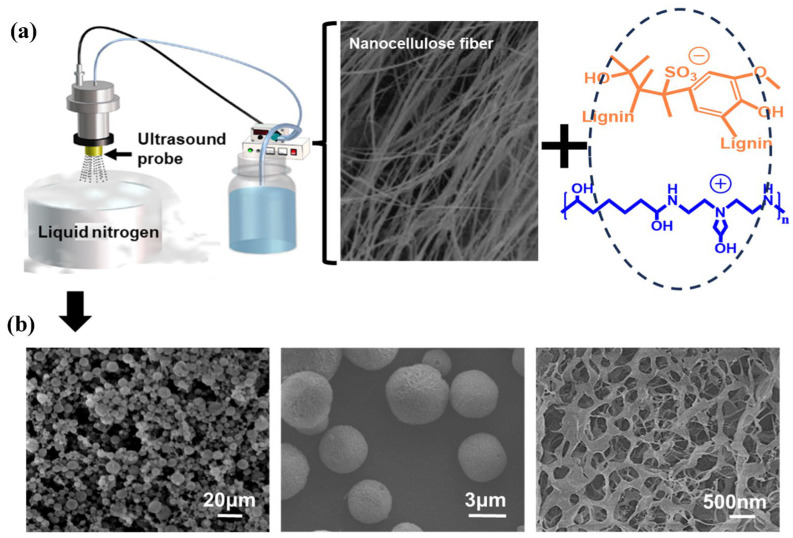
(**a**) Schematic diagram of ultrasonic nebulization for the preparation of the NLAG, including nanocellulose and lignin–PAE aqueous mixtures before nebulization, aerosol after nebulization, frozen particles in liquid nitrogen, and lyophilized NLAG. (**b**) NLAG morphologies captured at different magnifications.

The NLAG was prepared using an ultrasonic nebulizer (Figure 2a), where an aqueous suspension containing a mixture of nanocellulose and lignin–PAE resin nanoaggregates was nebulized into an aerosol and frozen in liquid nitrogen. Lignin–PAE resin nanoaggregates were prepared by mixing 2 wt.% lignin and 2 wt.% PAE. Then, they were mixed with a 2 wt.% nanocellulose fiber (NCF) suspension with no precipitate (Figure S1). After atomization, the ice template was removed by freeze-drying to avoid collapse of the nanocellulose backbone, in order to generate a high-porosity structure for the nanocellulose-mimetic aerogel. This freeze-drying technique has long been used to prepare many porous cellulose structures. After structure formation, the nanocellulose-mimetic aerogel was heated at 100 °C for 30 min in order to crosslink the PAE–LS and nanocellulose. Figure 2b shows the morphology of the nanocellulose aerogel at three different magnifications, indicating the particle size and microstructure of the nanocellulose aerogel. According to the results of scanning electron microscope calculations, most of the NLAG particles were in the range of 5–20 μm, and the overall particle size distribution was in the range of 1–25 μm. Upon magnification, it can be seen that the NLAG is a porous structure with a porosity of ~95%. However, the lignin–PAE adheres and binds to the nanocellulose backbone and does not cover the pores.

**Figure 3:**
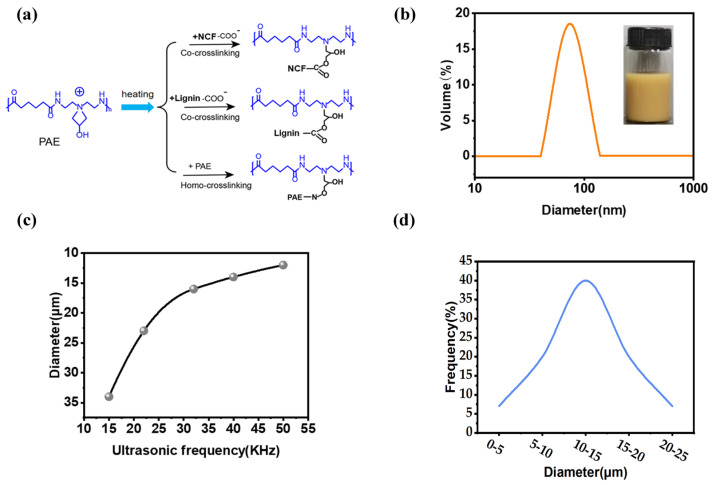
(**a**) Schematic of polymer interactions between lignin, PAE, and NFC. (**b**) Particle size distribution of lignin–PAE nanoclusters; a photograph of the lignin–PAE nanoclusters is shown in the inset. (**c**) Mean diameter variation with ultrasonic frequency. (**d**) Spherical diameter distribution from SEM images after the measurement of more than 500 NLAG particles.

Figure 3a shows a scheme of the reaction interactions associated with lignin, PAE, and NCF. The lignin–PAE resin nanoaggregates were filled into the cellulose nanofibers and formed a network through a combination of hydrogen bonding and covalent crosslinking. As reinforcing agents, lignin–PAE resin aerogels (average size ~80 nm; Figure 3b) were crosslinked with aerogels based on nanocellulose and lignin. This strategy has been reported for the preparation of cellulose papers and foams with good mechanical properties [15]. PAE resins can rapidly form covalent crosslinks at high temperatures (100 °C, 30 min) based on lyophilized structures, polymeric chain coupling between the azide group of PAE and OH, and interchain crosslinking of the azide group with the RCOO- component of lignin and cellulose [16], resulting in a very mechanically stable network. New peaks of the nanocellulose-mimetic aerogel were observed at 1733 and 1260 cm^−1^ (Figure S2). The ester bonds formed by crosslinking of the PAE resin during heating were confirmed by these bonds [17]. An endothermic peak (52–185 °C) appeared in the nanocellulose-mimetic aerogel during the first heating process (Figure S3), but there was no endothermic peak of the DSC curve during the second heating process. In conclusion, the successful establishment of the multi-crosslinked structure allows the nanocellulose aerogel to have good mechanical stability and to withstand the harsh environment that prevails during the water treatment process. The particle size changes in the aerogel after nebulization of the suspension were analyzed by scanning electron microscopy. It is worth noting that the particle size decreases in a quasi-linear manner with increasing ultrasonic output, i.e., a higher applied power produces smaller particles (Figure 3c). Under a high ultrasonic frequency, the particle size of the aerogel ranged between 1 and 25 μm (Figure 3d), the NLAG was observed to have a porous structure with a porosity of up to c. 95%, and the morphology of the aerogel showed a uniform mesh-like structure.

**Figure 4:**
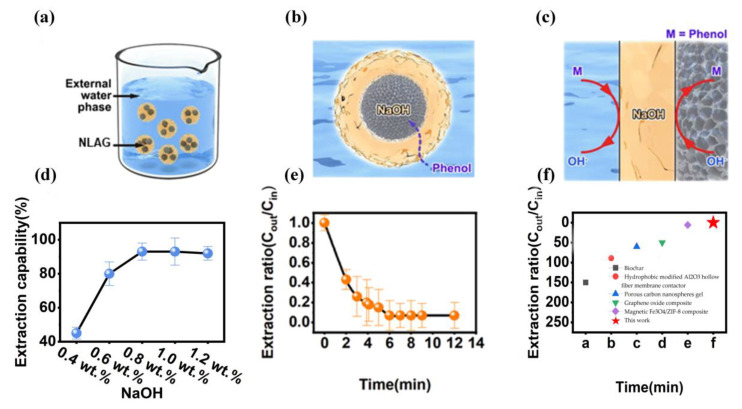
(**a**) Schematic diagram of the NLGA system dispersed in external water for phenol extraction. (**b**) Schematic diagram of the transport of phenol from external water to the NLAG, using NaOH as the stripper. (**c**) Extraction reactions occurring in the NLGA system (external water phase on the left; oil membrane phase in the middle; NLAG on the right). (**d**) The NLGA system with different NaOH concentrations in the oil-phase samples. (**e**) Extraction kinetics of phenol with an initial phenol concentration of 100 ppm, external water pH of 12, and an NLAG–water-to-be-treated volume ratio of 1:10; the NaOH concentration is 0.9 wt.%. (**f**) Comparison of the phenol extraction equilibrium time of the NLGA system with some previously reported materials.

**Figure 5:**
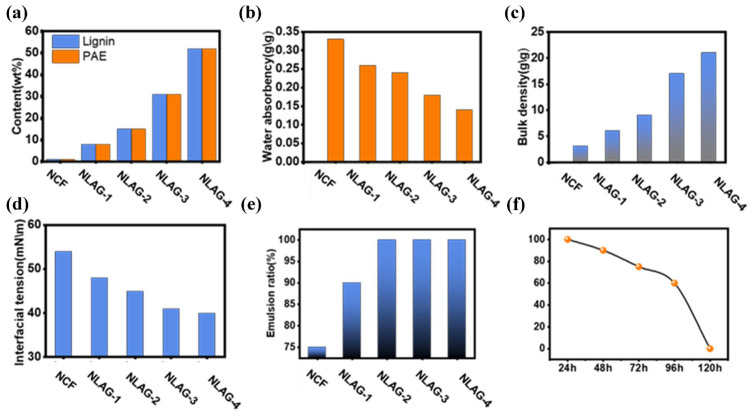
(**a**) NLAG samples with different lignin contents prepared by varying the amount of lignin–PAE in the NCF suspension. (**b**) Water absorption of different NLAG samples. (**c**) Bulk density values of different NLAG samples. (**d**) Interfacial tension values of water-absorbing NLAGs in the oil phase. (**e**) Emulsification rates of NLAG samples with different lignin contents. (**f**) Emulsification rates of the NLAG-2 samples as a function of time.

The biomimetic system was formed by dispersing the water-absorbing biomimetic aerogels in the oil phase via mechanical stirring (Figure 4a,b). Nanocellulose aerogels’ stability is affected by their surface wettability. The amphiphilic nature of cellulose and lignin has a specific affinity for oil and water, giving the nanocellulose-mimetic aerogels an extreme ability to adsorb polar and non-polar substances and promote stability at the oil–water interface (Figure 5c); because of these properties, nanopolymers of cellulose and lignin are widely used to make Pickering emulsions. However, cellulose is more hydrophilic than lignin due to its inherent molecular structure. Therefore, the ratio of nanocellulose to lignin composition in the nanocellulose-mimetic microware may exert a significant influence on the stability of the nanocellulose-mimetic system. To investigate this, nanocellulose-mimetic microware with different lignin–cellulose component ratios was prepared by varying the mixing ratio of nanocellulose to lignin–PAE resin prior to atomization. Generally, super-amphiphilic materials can be obtained by attaching hydrophilic and hydrophobic groups to the material, but the preparation of such materials often requires a tedious modification process. Therefore, both hydrophilic and lipophilic microdomains on the material surface are difficult to establish [18]. As components of NLAG, lignin and cellulose are amphiphiles that contribute to the stability of the oil–water interface in addition to improving the mechanical stability. Due to its inherent molecular structure, cellulose is more hydrophilic than lignin [18]; therefore, the compositional ratio of nanocellulose to lignin in NLAGs may have a significant effect on the stability of NLGA systems. For this purpose, NLAGs with different lignin–cellulose composition ratios were prepared by varying the mixing ratio of nanocellulose and lignin–PAE resin before atomization (Figure 5a). Furthermore, Figure 4b demonstrates that the water absorption of the aerogels decreased with increasing lignin content, and the stacking density results indicate that the empty space was occupied by the lignin–PAE resin (Figure 5c). The results of the interfacial tension test are shown in Figure 4d, where the oil–water interfacial tension of the surface interface of the NLAG was measured and the value decreased from 55 mN/m to 40 mN/m, corresponding to a sample without lignin and one with a lignin content of 50 wt.%, respectively. This finding indicates that the stability of the system was improved by the introduction of lignin. In addition, the emulsion fraction (Figure 5e) confirmed that the incorporation of lignin could facilitate the construction of a more stable NLGA system. Furthermore, the NLGA system produced by NLAG-2 could be maintained for at least 24 h (Figure 5f), which was enough time for the extraction applications.

In practical experiments, phenol pollutants were extracted from water using the abovementioned BSM, consisting of an exfoliant (NaOH) dissolved in the organic phase within an NLAG. During the extraction process, the biofilm system was poured into the water to be treated while stirring at 500 rpm to break the biofilm system into small droplets of a few centimeters in size (Figure 4a). Using the droplets of the biofilm system for phenol extraction, phenol was transported from the external water to the internal NLAG (Figure 4b). Meanwhile, at the interface of the NLAG, the phenol was stripped by NaOH and converted into a new compound that could not reversibly penetrate the NLAG. The dual-interface reaction of the membrane phase with NaOH as an extractant is shown in Figure 5c, and the NaOH concentration of ≥8 wt.% shows a high extraction performance toward phenol. The removal capacity of the biofilm system was monitored by determining the phenol concentration in the water (Figure 4e). For the phenol profile, the removal rate of ambient phenol was close to 93%. In practical applications, the extraction capacity of the biofilm system was investigated for different external hydroxide ion concentrations, different volume ratios of the biofilm system to the external water, and different external water pH values (Figure S4). It is noteworthy that the liquid-membrane-based extraction of the biofilm system allows for high-speed mass transfer. As a result, the equilibrium time of the biofilm system was found to be much higher than that reported for phenol extraction sorbents (Figure 4f) [19,20,21,22,23].

**Figure 6:**
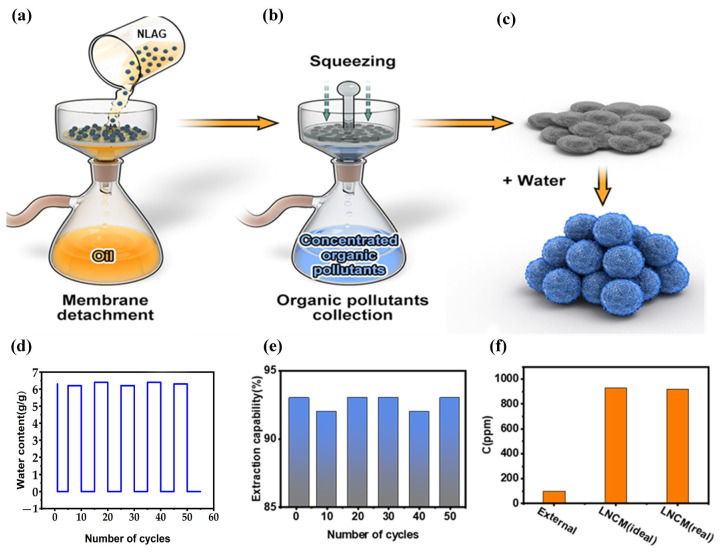
(**a**) The detachment of NLGA by filtration. (**b**) Collection of concentrated organic matter in the NLAG by extrusion. (**c**) The compressed NLAG can be regenerated by suction for recycling. (**d**) NLAG stripper storage capacity after 50 suction/dewatering cycles. (**e**) NLAG extraction capacity after 50 cyclic operations. (**f**) Phenol concentration in the initial external water prior to extraction, and phenol concentration in the NLAG after extraction.

Although emulsion membrane methods are very effective and have been successfully studied for decades, their commercial application for the removal of waste contaminants is limited by the difficulty of breaking emulsions after extraction. In emulsion membrane processes, chemical breaking methods are limited by the difficulty of reusing the oil phase. The NLGA system can be easily separated by the filtration process (Figure 6a). In addition, the rapid shape-recovery properties of NLAGs are highly desirable. The process of collecting concentrated phenol ions and the regeneration of repeated extractions is shown in Figure 6b. The shape-recovery properties are shown in Figure 6c. The volume of the original water-absorbing NLAG was set to 10 mL, and the volume of the compressed aerogel was reduced to 2.5 mL after dehydration; the aerogel rapidly recovered to its original shape within 1 s upon reabsorption of water. This water-sensitive shape recovery far exceeded that reported for cellulose foam [24]. Concentrated substances such as phenol in the aerogel were easily squeezed and rinsed, indicating that the aerogel can be fully reused. The lignin on the fiber surface gives the fiber elasticity and partially prevents it from swelling. In addition, co-crosslinking between the cellulose and the lignin–PAE resin complexes imparts toughness and flexibility to the network. In an aerogel-based separation system, both the aerogel and the organic phase can be recovered and reused by simple filtration.

In the experiments, the recovered aerogels were regenerated, washed, and reused 50 times (Figure 6d), and the phenol extraction ability of the NLGA system did not degrade after multiple cycles. The results showed that the crosslinked aerogels could be well preserved during the regeneration and separation process, and the morphology of the aerogels also remained significantly unchanged after 50 regeneration cycles. As shown in Figure 6e, the aerogels still exhibited high effective extraction efficiency after multiple regeneration cycles, indicating that the cellulose nanofiber aerogels are highly reusable in W/O/W membrane separation systems. Extraction from the NLGA system can yield high concentrations of phenol, and squeezing phenol out of the aerogel allows for the easy collection of new phenol. As shown in Figure 6f, the concentration of phenol in the NLAG is about nine times higher than the initial external water concentration. Moreover, this value is almost equal to the ideal phenol concentration calculated from the reduction of phenol in external water, indicating that the interaction of phenol with the NLAG is neglected. In conclusion, the separability of this NLGA system allows for a sustainable re-extraction of phenol rather than a high-performance extraction, making it an efficient process for extraction applications.

**Figure 7:**
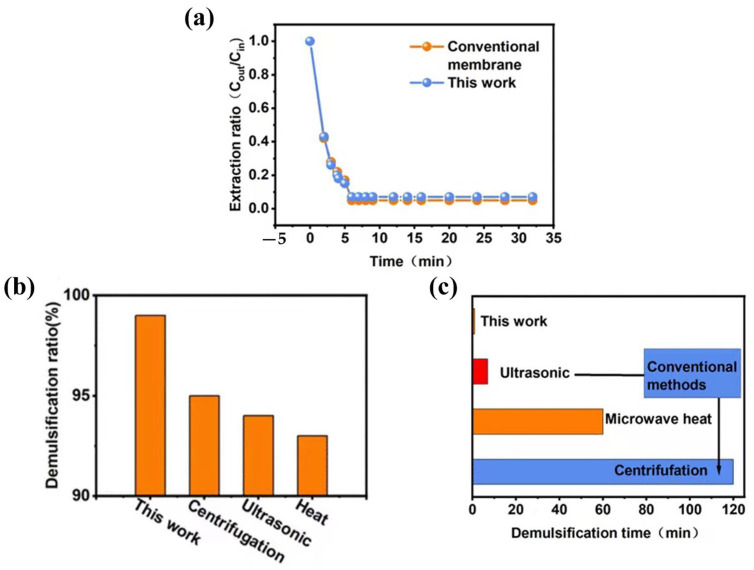
(**a**) The extraction capability of NLGA and the conventional emulsion liquid membrane. (**b**) The demulsification times of NLGA compared with conventional demulsification methods used in conventional emulsion liquid membranes. (**c**) The demulsification ratios of our work compared with other methods used in the conventional emulsion liquid membranes.

NLGAs are easier to demulsify than conventional emulsion membrane systems, even though they are both based on liquid–liquid transfer for extraction (Figure 7). Most physical emulsion-breaking methods, such as centrifugation and high-voltage electric field methods, are expensive, inefficient, and energy intensive [25,26]. The extraction capacity of NLGAs is comparable to that of conventional emulsion membrane systems (without NLAG) (Figure 7a). However, as shown in Figure 7c, the conventional membrane demulsification process takes anywhere from 7 to 120 min, whereas our NLGA passes through filtration in only 1 min. In addition, the demulsification performance (demulsification rate) of the conventional method (Figure 7b) was also unsatisfactory due to the difficulty of completely breaking down the very stable emulsion [25,27,28]. In conclusion, not only can NLGA be used to easily prepare and efficiently extract phenol (Figure 5), but it is also able to easily break the emulsion compared to conventional emulsion membrane systems.

## 3. Conclusions

In conclusion, we successfully prepared NLGAs from cellulose nanofibers using ultrasonic atomization. Most of these aerogels measured between 1 μm and 25 μm and were highly porous and ultralight. The prepared aerogels were resistant to harsh environments through covalent crosslinking. All of these features demonstrate the promising application of biomimetic cellulose nanofiber aerogels, especially as W/O/W systems for the extraction of phenol from water. By simple low-shear stirring, the saturated aerogel can be well dispersed in the organic phase, forming a stable suspension. In addition, the aerogel can be easily separated by filtration and reused in a new separation system, without the need for a complex emulsion-breaking process. These results show that natural green aerogels have significant potential for the separation and extraction of organic contaminants such as phenols.

## 4. Materials and Methods

### 4.1. Materials

Sodium lignosulfonate (LS) was purchased from Sinopharm Chemical Reagent Co. (Shanghai China); nanocellulose (NCF) was purchased from Qihong Chemical Reagent Co. (Guilin China); PAE resin was purchased from Shandong Xindi Co. (Jining China); lignin, silicone oil, Span-80, sodium hydroxide, and phenol (all chemically pure) were purchased from Sinopharm Chemical Reagent Co., Ltd (Shanghai China).

### 4.2. Preparation of Aerogels and the Biomimetic Membrane

The stabilized LS–PAE complex (pH∼5.4, zeta potential∼24 mV) was obtained by mixing LS (2 wt.%) and PAE (2 wt.%) at a 1:1 v/v ratio; 50 mL of LS–PAE was added to 50 mL of NCF suspension (2 wt.%) and mixed homogeneously for 30 min under magnetic stirring at 500 rpm. Afterwards, the suspension was nebulized using a non-ultrasonic nebulizer at a rate of 10 mL/min. To produce crosslinked aerogels, the dried samples were then cured in a vacuum oven at 120 °C for 3 h.

The biomimetic membrane was prepared by dispersing the aerogel and absorbed NaOH solution (0.8 wt.%). In detail, excess 0.8 wt.% NaOH was mixed with 20 mL (bulk volume) of aerogel in a cylinder, and then the cylinder was stirred manually for 10 min until no excess liquid flowed out. Then, 20 mL of aerogel + NaOH (bulk volume) was dispersed in 40 mL of silicone oil with 0.4 mL of Span-80 and then homogenized using a homogenizer at 8000 rpm for 5 min to generate the biomimetic membrane (NLGA).

### 4.3. Extraction and Recovery of Organic Pollutants

The phenol solution for this study was synthetically prepared at the desired concentrations, which then allowed for the extraction of the NLGA. In detail, the NLGA was exposed to a phenol solution at a concentration of 100 mg L^−1^ and stirred at a low rate of 200 rpm. The absorbance was measured using a UV–visible spectrophotometer (UV757CRT/PC) at 287 nm, and the concentration of phenol in the separated external phase was then calculated.

Once the extraction step was complete, the membrane and the external phase were separated by sedimentation. The oil phase containing saturated aqueous aerogel was filtered to collect nanocellulose microspheres. The filtrate, containing the organic oil, emulsifier, and carrier, can be reused directly. The filtered saturated aqueous aerogels were regenerated by extrusion from the phenol–sodium solution and reused for a new batch of experiments.

### 4.4. Characteristic Measurement

FTIR spectra were acquired using a Bruker Vertex 80 V spectrometer with a detector resolution of 4 cm^−1^ and a range of 600 to 4000 cm^−1^, with 32 scans per sample. Calorimetric measurements were conducted on a differential scanning calorimeter (DSC, Netzsch 214 Polyma). In the first run, the sample was cooled to 0 °C and then heated to 250 °C at a rate of 10 °C/min. The first run’s steps were repeated for the second run. Morphological characterization was performed using a scanning electron microscope (SEM, Hitachi SU8010). The zeta potential was measured on a Micrometrics^®^ NanoPlus-2 device (Shanghai, China) according to the principle of laser Doppler electrophoresis. A surface tension meter (BZY-3B/4B) was used for morphological characterization. The phenol concentration (wavelength: 287 nm) was determined using a UV–visible spectrophotometer (UV757CRT/PC).

## Data Availability

The data presented in this study are openly available in article.

## Supplementary Materials

The following supporting information can be downloaded at: https://www.mdpi.com/article/10.3390/gels10010059/s1, Figure S1: the lignin–PAE/CNF mixture suspension used for the ultrasonic atomization.; Figure S2: FTIR spectra of pure nanocellulose and NLAG; Figure S3: DSC curves of the NLAG with two temperature increases; Figure S4: Extraction capacity of BSM.

## References

[1] Smriti S.A., Haque A.N.M.A., Khadem A.H., Siddiqa F., Rahman A.N.M.M., Himu H.A., Farzana N., Islam M.A., Naebe M. (2023). Recent developments of the nanocellulose extraction from water hyacinth: A review. Cellulose.

[2] Shanmugarajah B., Chew I.M., Mubarak N.M., Choong T.S., Yoo C., Tan K. (2019). Valorization of palm oil agro-waste into cellulose biosorbents for highly effective textile effluent remediation. J. Clean. Prod..

[3] Feng Y., Qiu X., Tao Z.E.Z., Song J., Dong Y., Liang J., Li P., Fan Q. (2022). Oxygen-containing groups in cellulose and lignin biochar: Their roles in U(VI) adsorption. Environ. Sci. Pollut. Res..

[4] Ramirez-Muñoz A., Pérez S., Muñoz-Saldaña J., Flórez E., Acelas N. (2021). Eco-friendly materials obtained through a simple thermal transformation of water hyacinth (*Eichhornia crassipes*) for the removal and immobilization of Cd^2+^ and Cu^2+^ from aqueous solutions. Environ. Nanotechnol. Monit. Manag..

[5] Amalina F., Razak A.S.A., Krishnan S., Zularisam A.W., Nasrullah M. (2022). Water hyacinth (*Eichhornia crassipes*) for organic contaminants removal in water—A review. J. Hazard. Mater..

[6] Righini G. (2018). Glassy Microspheres for Energy Applications. Micromachines.

[7] Salviati S., Carosio F., Cantamessa F., Medina L., Berglund L.A., Saracco G., Fina A. (2020). Ice-templated nanocellulose porous structure enhances thermochemical storage kinetics in hydrated salt/graphite composites. Renew. Energ..

[8] Cao N., Lyu Q., Li J., Wang Y., Yang B., Szunerits S., Boukherroub R. (2017). Facile synthesis of fluorinated polydopamine/chitosan/reduced graphene oxide composite aerogel for efficient oil/water separation. Chem. Eng. J..

[9] Zhang F., Li Y., Cai H., Liu Q., Tong G. (2020). Processing nanocellulose foam into high-performance membranes for harvesting energy from nature. Carbohydr. Polym..

[10] Zhang F., Lan X., Peng H., Hu X., Zhao Q. (2020). A “Trojan Horse” Camouflage Strategy for High-Performance Cellulose Paper and Separators. Adv. Funct. Mater..

[11] Kanomata K., Fukuda N., Miyata T., Lam L.P.Y., Takano T., Tobimatsu Y., Kitaoka T. (2019). Lignin-Inspired Surface Modification of Nanocellulose by Enzyme-Catalyzed Radical Coupling of Coniferyl Alcohol in Pickering Emulsion. ACS Sustain. Chem. Eng..

[12] Bai L., Greca L.G., Xiang W., Lehtonen J., Huan S., Nugroho R.W.N., Tardy B.L., Rojas O.J. (2018). Adsorption and Assembly of Cellulosic and Lignin Colloids at Oil/Water Interfaces. Langmuir.

[13] Nair S.S., Chen H., Peng Y., Huang Y., Yan N. (2018). Polylactic Acid Biocomposites Reinforced with Nanocellulose Fibrils with High Lignin Content for Improved Mechanical, Thermal, and Barrier Properties. ACS Sustain. Chem. Eng..

[14] Zhang D., Jiang Q., Liang D., Huang S., Liao J. (2021). The Potential Application of Giant Reed (*Arundo donax*) in Ecological Remediation. Front. Environ. Sci..

[15] Yin H., Zheng P., Zhang E., Rao J., Lin Q., Fan M., Zhu Z., Zeng Q., Chen N. (2020). Improved wet shear strength in eco-friendly starch-cellulosic adhesives for woody composites. Carbohydr. Polym..

[16] Sirviö J.A., Visanko M. (2020). Lignin-rich sulfated wood nanofibers as high-performing adsorbents for the removal of lead and copper from water. J. Hazard. Mater..

[17] Qiao L., Li S., Li Y., Liu Y., Du K. (2020). Fabrication of superporous cellulose beads via enhanced inner cross-linked linkages for high efficient adsorption of heavy metal ions. J. Clean. Prod..

[18] Song Y., Zhou J., Fan J.B., Zhai W., Meng J., Wang S. (2018). Hydrophilic/Oleophilic Magnetic Janus Particles for the Rapid and Efficient Oil–Water Separation. Adv. Funct. Mater..

[19] Hairuddin M.N., Mubarak N.M., Khalid M., Abdullah E.C., Walvekar R., Karri R.R. (2019). Magnetic palm kernel biochar potential route for phenol removal from wastewater. Environ. Sci. Pollut. Res..

[20] Salari M., Dehghani M.H., Azari A., Motevalli M.D., Shabanloo A., Ali I. (2019). High performance removal of phenol from aqueous solution by magnetic chitosan based on response surface methodology and genetic algorithm. J. Mol. Liq..

[21] Zhang J., Qin L., Yang Y., Liu X. (2021). Porous carbon nanospheres aerogel based molecularly imprinted polymer for efficient phenol adsorption and removal from wastewater. Sep. Purif. Technol..

[22] Liu Y., Huang Y., Chen G., Huang J., Zheng J., Xu J., Liu S., Qiu J., Yin L., Ruan W. (2018). A graphene oxide-based polymer composite coating for highly-efficient solid phase microextraction of phenols. Anal. Chim. Acta.

[23] Qu Y., Qin L., Liu X., Yang Y. (2022). Magnetic Fe_3_O_4_/ZIF-8 composite as an effective and recyclable adsorbent for phenol adsorption from wastewater. Sep. Purif. Technol..

[24] Zhang W., Zhang Y., Lu C., Deng Y. (2012). Aerogels from crosslinked cellulose nano/micro-fibrils and their fast shape recovery property in water. J. Mater. Chem. A.

[25] Zheng X., Ying Z., Cui J., Wang B., Chen J., Zhang Q. (2017). Simultaneous Dewatering and Recovering Oil from High-Viscosity Oily Sludge through the Combination Process of Demulsification, Viscosity Reduction, and Centrifugation. Energy Fuels.

[26] Peng Y., Liu T., Gong H., Zhang X. (2016). Review of the Dynamics of Coalescence and Demulsification by High-Voltage Pulsed Electric Fields. Int. J. Chem. Eng.

[27] Yang X.g., Tan W., Tan X.f. (2009). Demulsification of Crude Oil Emulsion via Ultrasonic Chemical Method. Pet. Sci. Technol..

[28] Faizullayev S., Adilbekova A., Kujawski W., Mirzaeian M. (2022). Recent demulsification methods of crude oil emulsions–Brief review. J. Pet. Sci. Eng..

