# Supplementary material for: A Biomimetic Lignocellulose Aerogel-Based Membrane for Efficient Phenol Extraction from Water
Source: Gels. 2024 Jan 12;10(1):59. doi: 10.3390/gels10010059 (PMC10815555; doi:10.3390/gels10010059)
Supplement: Supplementary file 1 [file gels-10-00059-s001.zip › gels-2755618-supplementary.pdf]

Supplementary Material

A Biomimetic Lignocellulose Aerogel-Based Membrane for Efficient Phenol Extraction from Water

Peipei Liu, Chunling Zheng, Zhong Yao * and Fang Zhang *

College of Food Science and Light Industry, Nanjing Tech University, Nanjing 211800, China; lppliupp@163.com (P.L.); zhengchunling@njtech.edu.cn (C.Z.)

* Correspondence: yaozhong@njtech.edu.cn (Z.Y.); zfygjy@163.com (F.Z.)

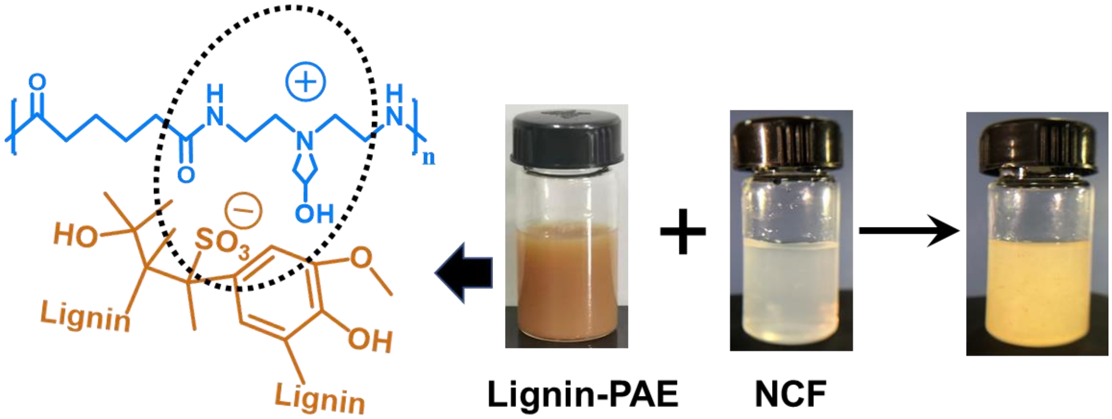

Figure S1. The lignin-PAE/CNF mixture suspension used for the ultrasonic atomization. Left the complex mechanism between lignin and PAE. Lignin-PAE was prepared by mixing equal volume of the 2 wt.% lignin and 2 wt.% PAE, CNF concentration was 2 wt.%.

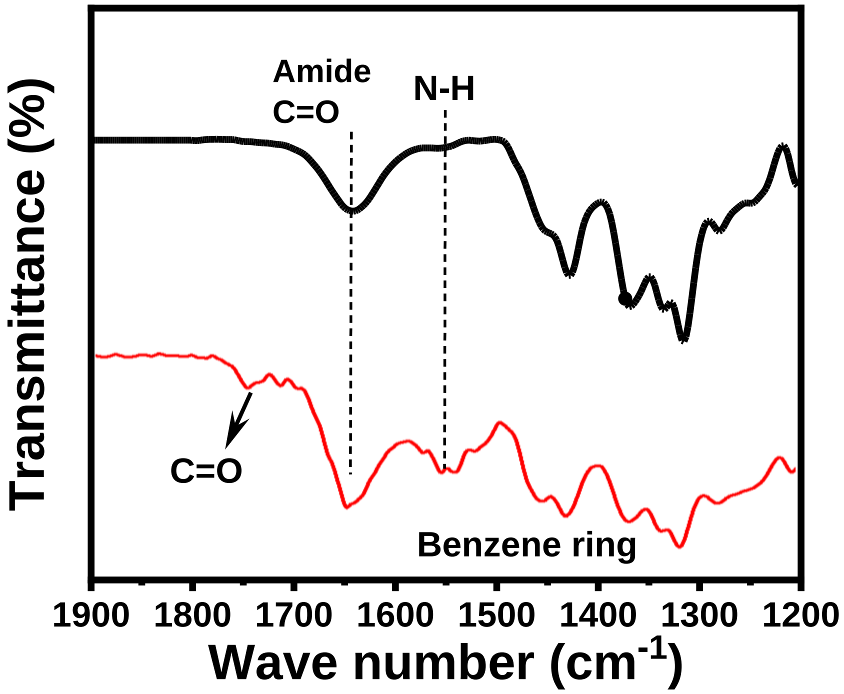

Figure S2. FTIR spectra of pure nanocellulose and NALG.

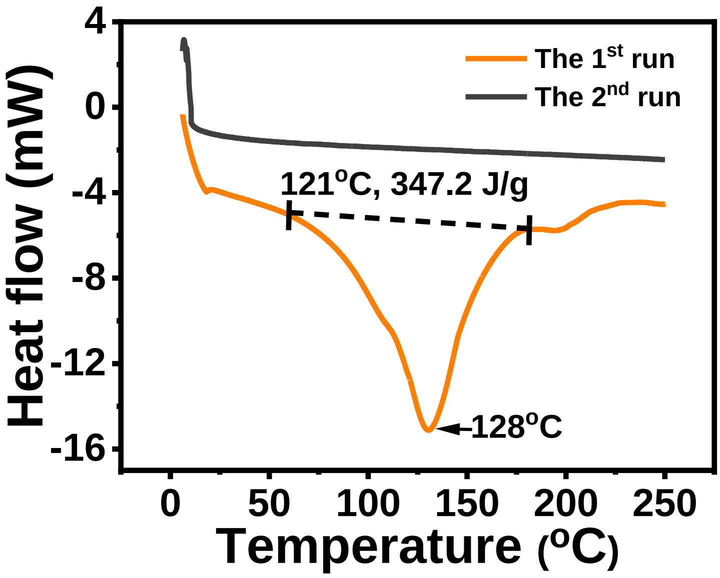

Figure S3. DSC curves of the NALG with two temperature increases.

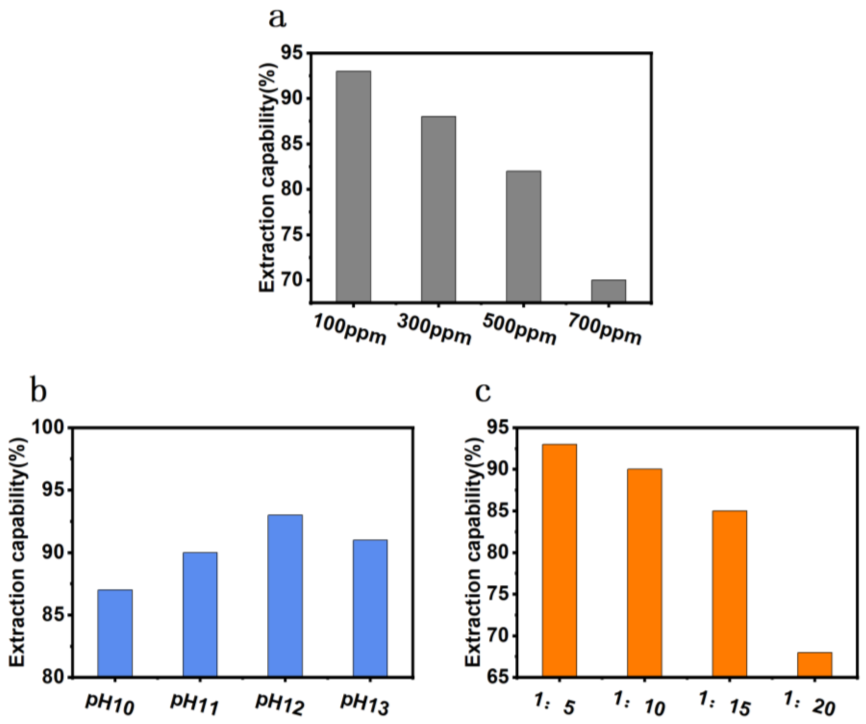

Figure S4. Extraction capacity of BSM for (**a**) Different external phenol concentrations with a volume ratio of 1:10 and a pH of 12 (**b**) Different external water pHs with an external phenol concentration of 100 ppm and a volume ratio of 1:10 (**c**) Different external water volume ratios with an applied phenol concentration of 100 ppm and an applied water pH of 12.
